# Computed Tomography Severity Scoring on High-Resolution Computed Tomography Thorax and Inflammatory Markers With COVID-19 Related Mortality in a Designated COVID Hospital

**DOI:** 10.7759/cureus.24190

**Published:** 2022-04-16

**Authors:** Bukke Ravindra Naik, Anil K Sakalecha, Sunil B N, Chaithanya A, Mahima Kale R, Kalathuru Uhasai

**Affiliations:** 1 Radiology, Sri Devaraj Urs Medical College, Kolar, IND; 2 Radio-Diagnosis, Sri Devaraj Urs Medical College, Kolar, IND; 3 Community Medicine, Sri Devaraj Urs Medical College, Kolar, IND

**Keywords:** hrct of chest, rt-pcr, pneumonia, research in emergency medicine, ctss, covid-19

## Abstract

Introduction

Radiological Society of the Netherlands introduced the coronavirus disease 2019 (COVID-19) Reporting and Data System (CO-RADS) and the corresponding CT severity score (CTSS) to diagnose COVID-19 severity. However, data regarding the same is very limited.

Objectives

The objective of this study was to correlate the computed tomography severity scoring (CTSS) on high-resolution computed tomography (HRCT) thorax and inflammatory markers with COVID-19 related mortality.

Methods

A retrospective observational study was conducted in a tertiary center between June 2020 to May 2021 among 2343 adult patients at the department of radio-diagnosis with suspected and confirmed COVID-19 cases who received an HRCT thorax. Data was collected retrospectively from the records regarding age, sex, and information regarding inflammatory markers such as C-reactive protein (CRP), ferritin, lactate dehydrogenase (LDH), erythrocyte sedimentation rate (ESR), D-dimer, and neutrophil-to-lymphocyte ratio. Information on HRCT thorax of patients was reviewed for radiological suspicion of COVID-19 related lung changes using CO-RADS scoring and severity of lung involvement using CT-severity scoring. Data was analyzed using SPSS version 22 (IBM Inc., Armonk, New York).

Results

The mean age was 51.69 ± 16.02 years, and most of the study participants were male (1592, 67.95%). The majority (999, 42.64%) had diabetes as a comorbidity. The reverse transcription polymerase chain reaction (RT-PCR) test was positive in 1571 (67.05%) participants. The majority (1571, 67.05%) had a CO-RADS score of six, and only 150 (6.40%) had CO-RADS score of four. The CT severity score was normal in 147 (6.27%), mild in 724 (30.90%), moderate in 903 (38.54%), and severe in 569 (24.29%) participants. The CRP levels were moderate in 1200 (51.22%) and severe in 428 (18.27%) participants. The mean ferritin, D-dimer and interleukin-6 (IL-6) levels were 321.83 ± 266.42 ng/ml, 1.51 ± 0.85mg/l, and 323.05 ± 95.52pg/ml, respectively. The mean length of hospital stay was 10.25 ± 6.52 days. Most of them (1926 out of 2343, 82.20%) survived, and nearly 417 out of 2343 (17.80%) died. Out of 2343, 569 participants had severe CT severity scores, out of which 205 (36.03%) died, and 364 (63.97%) participants survived.

Conclusion

A positive correlation was found between CT severity scoring on HRCT thorax and inflammatory markers with COVID-19 related mortality and can be used in early diagnosis and timely management of COVID-19 positive patients.

## Introduction

Currently, there is a health emergency being faced globally due to the novel coronavirus, which was initially reported in 2019 in Wuhan, China. Thorough knowledge of the chest imaging findings of COVID-19 has already been established to make accurate and early diagnoses of this deadly disease [1]. However, the disease burden on a patient's life, including its mortality and morbidity, can sometimes be difficult to predict, resulting in asymptomatic to severe life-threatening clinical outcomes. The predominant presentation of the COVID-19 infection is characterized by high temperature and cough [2]. At present, the diagnostic strategy is based on the combination of a history of exposure, clinical characteristics, reverse transcription polymerase chain reaction (RT-PCR) assay followed by a chest X-Ray (CXR) and high-resolution computed tomography (HRCT) thorax [3].

The primary mode of transmission is in the form of respiratory droplets. The majority of deaths due to COVID-19 can be attributed to lower respiratory infection, which also seems to be the common manifestation of the disease. Clinically these patients present with flu-like symptoms, dyspnoea, and shortness of breath. Data on a small number of hospitalized COVID-19 patients showed pneumonia on CT scan, eventually progressing to acute respiratory distress syndrome (ARDS) in 17-29% of cases [4]. A study by the Chinese Centre for Disease Control and Prevention [5] found nearly 44,500 confirmed COVID-19 cases and 81% cases had a mild infection (no or mild pneumonia), 14% had severe disease (clinically or >50% lung involvement on imaging), 5% had multiorgan failure, and the overall mortality was 2.3%. Most of the severe COVID-19 patients show abnormal imaging. A recent study found 97% sensitivity and 25% specificity for CT scans when compared to RT-PCR testing as the reference standard [6].

The HRCT chest examines the severity of lung involvement, complications, and helps in providing a treatment plan for patients based on disease severity and is currently used as a diagnostic tool for COVID-19 patients. Bilateral multifocal peripheral ground-glass opacities are typical findings of COVID-19 pneumonia on the HRCT chest [7]. However, a regular HRCT study cannot rule out the possibility of the disease; it only suggests that there is no involvement of the lung parenchyma at the time of the study. HRCT has a sensitivity of 89% and specificity of 68% and is used as a first-line screening investigation for COVID-19 in emergency and hospital settings, compared to RT-PCR. Hence, chest CT or HRCT combined with clinical and laboratory evaluation can be used as the gold standard for diagnosing COVID-19 [8,9]. Yusuf et al. [10], in Pakistan, found hallmark findings on non-contrast HRCT chest scans regardless of a negative RT-PCR assay among 48 COVID-19 positive patients. Another retrospective study by Saeed et al. [11] in UAE found that the 25-point CT severity score correlates well with the COVID-19 clinical severity in predicting COVID-19 disease outcome and significantly correlates with lab tests and oxygen requirements.

The existing literature worldwide has explored pulmonary involvement in the chest CT images. Further, hardly any studies correlate this CT severity scoring on HRCT and inflammatory markers with COVID-19 related mortality in developing countries like India. Hence the present study was undertaken to determine the correlation of CT severity scoring on HRCT thorax and inflammatory markers with COVID-19 related mortality in a designated COVID hospital. The study aims to evaluate CT severity scoring on HRCT thorax and inflammatory markers in patients with clinical symptoms and suspicion of COVID-19 disease and patients who were confirmed to the COVID-19 positive on the RT-PCR test, and to correlate this CT severity scoring on HRCT thorax and inflammatory markers with COVID-19 related mortality. 

## Materials and methods

A retrospective observational study was conducted among 2343 COVID-19 positive and suspected patients referred for HRCT thorax to the imaging unit of the department of radio-diagnosis in a designated COVID hospital - RL Jalappa Hospital in Kolar, India. The study was conducted from June 2020 to May 2021. Institutional Ethics committee approval was taken before the study was started. As the study was retrospective, the informed consent was waived off by the ethics committee. To avert any potential breach of confidentiality, the patient's names were not revealed.

Sample size calculation

The sample size was estimated by using the proportion of chest CT findings related to a study by Hul et al. [12] using the following formula:

Sample size = z1-α/22 p(1-p)/d2, where z1-α/22= 1.96 at 5% alpha error

p= expected proportion in population-based on previous studies (0.55), d= absolute error or precision (10%)

Using the above values at a 95% confidence level, a sample size of 2343 was included and evaluated as per study objectives. For the feasibility of the study, a universal sampling method was used to select the required sample size.

Inclusion and exclusion criteria

Patients with clinical symptoms and suspicion of COVID-19 disease who underwent HRCT thorax and patients diagnosed to be COVID‑19 positive by RT‑PCR testing and underwent HRCT thorax were included in the study. Pregnant and lactating patients, and debilitated patients requiring ventilator support who were not in a position for scanning were excluded from the study.

Data collection

Data was collected retrospectively from the age, sex, travel, and exposure history records. Information regarding inflammatory markers such as C-reactive protein (CRP), ferritin, lactate dehydrogenase (LDH), erythrocyte sedimentation rate (ESR), D-dimer, and neutrophil-to-lymphocyte ratio was also collected from the records. Information on HRCT thorax of patients was reviewed for radiological suspicion of COVID-19 related lung changes using COVID-19 Reporting and Data System (CO-RADS) scoring and severity of lung involvement using CT-severity scoring.

The procedure of HRCT

HRCT thorax was performed using the SOMATOM Emotion 16 slice computed tomography machine (Siemens Healthineers, Erlangen, Germany). The procedure did not involve the use of any intravenous contrast agents. Patients were asked to lay in the supine position and were then scanned in the craniocaudal direction from the apex of the lungs to the diaphragm and were instructed to hold their breath for 7-8 seconds during the scan. The technical parameters were kept at 120 kVp, 300 mAs, and a rotation time of 0.5 sec. A 2 mm scanning slice thickness and 10mm intersection interval were maintained. After automatic reconstruction, the interval was later reduced to 0.5 mm. After each suspected COVID-19 patient was scanned, the scanner was adequately disinfected. The HRCT images which were reconstructed were then transferred to the Myrian 3D workstation. The images were viewed in coronal, sagittal, and axial views for assessing lung and mediastinal windows. Two reviewers analyzed the scan, and the radiological suspicion of COVID-19 disease was made based on the CO-RADS scoring system. The severity of lung involvement was described based on total severity scoring on a scale of 25 points. Evaluation of the scan was based on the severity of lung involvement and graded using CT severity scoring.

Operational definitions of CO-RADS scores are explained in Table 1 [13].

**Table 1:** The CO-RADS categories used for CT scoring CO-RADS: coronavirus disease 2019 Reporting and Data System, RT-PCR: reverse transcription polymerase chain reaction, SARS-CoV-2: severe acute respiratory syndrome coronavirus 2

CO-RADS score	Level of suspicion	Findings
CO-RADS 0	Not interpretable	Scan technically insufficient for assigning a score
CO-RADS 1	Very low	Normal or non-infectious
CO-RADS 2	Low	Typical for other infections but not COVID-19
CO-RADS 3	Equivocal/unsure	Features compatible with COVID-19, but also other diseases
CO-RADS 4	High	Suspicious for COVID-19
CO-RADS 5	Ver high	Typical for COVID-19
CO-RADS 6	Proven case	RT-PCR positive for SARS-CoV-2

Based on the above findings, each patient was categorized as CO-RADS 0, 1, 2, 3, 4, 5, or 6. 

The CT severity score index is an estimation of pulmonary/lung involvement by COVID-19. Each of the five lung lobes (three right and two left) were scored visually and given a scoring from 1 to 5 (Table 2, 3)

**Table 2:** CT severity score index

% Involvement (single lobe)	Score
0-5 % lung involvement	1
5-25 % lung involvement	2
25-50 % lung involvement	3
50-75 % lung involvement	4
75-100 % lung involvement	5

**Table 3:** CT severity score calculation criterion

CT severity	SCORE
Mild	< 8
Moderate	9 - 15
Severe	> 15
Total score	~ 25

Statistical analysis

COVID-related mortality was considered as the primary outcome variable. Descriptive statistics were used to analyze data by the study's objectives. Data was expressed as the mean, 95% confidence interval (CI; lower and upper bounds), median, minimum and maximum, and percentage, where appropriate. Categorical outcomes were compared between study groups using the Chi-square test. A p-value < 0.05 was considered statistically significant. Data was analyzed by using coGuide V.1.0.3 Statistical Software (CoGuide, Bangalore, India) [14].

## Results

A total of 2343 participants were included in the final analysis. The demographic and comorbidities data is presented in Table 4.

**Table 4:** Summary of baseline parameters (N=2343) CKD: chronic kidney disease, PTB: pulmonary tuberculosis

Parameter	Summary
Mean age (in years)	51.69 ± 16.02 (ranged 18 to 96)
Gender	
Male	1592 (67.95%)
Female	751 (32.05%)
Comorbidities	
Diabetes	999 (42.64%)
Hypertension	844 (36.02%)
CKD	109 (4.65%)
Bronchial asthma	61 (2.60%)
PTB	52 (2.22%)
Other	241 (10.29%)

The mean age was 51.69 ± 16.02 years, and most of the study participants were male, 1592 (67.95%). The majority of 999 (42.64%) were suffering from diabetes, 844 (36.02%) from hypertension, and only 61 (2.60%) participants suffered from bronchial asthma.

The RT-PCR test was positive in 1571 (67.05%) participants with a CO-RADS score of 6. In the rest of the cases, the majority (622, 26.55%) had a CO-RADS score of 5, and only 150 (6.40%) had a CO-RADS score of 4. The CT severity score was normal in only 147 (6.27%) participants, mild in 724 (30.90%) participants, moderate in 903 (38.54%) participants, and severe in 569 (24.29%) participants. The CRP levels were moderately increased in 1200 (51.22%) participants and severely increased in 428 (18.27%) participants. The mean ferritin level was 321.83 ± 266.42 ng/ml, lactate dehydrogenase (LHD) levels were 471.55 ± 290.58 U/L, erythrocyte sedimentation rate (ESR) was 53.6 ± 44.03 m/h, D-dimer was 1.51 ± 0.85 mg/l, interleukin-6 (IL-6) was 323.05 ± 95.52 pg/ml, the neutrophil-to-lymphocyte ratio was 8.4 ± 5.46 pg/ml, and the mean length of hospital stay was 10.25 ± 6.52 days (ranging from 1 to 41). The majority (1926, 82.20%) of the participants survived, and nearly 417 (17.80%) participants died due to COVID-19 related complications (Table 5).

**Table 5:** Summary of outcome parameters (N=2343) RT-PCR: reverse transcription polymerase chain reaction, LDH: lactate dehydrogenase, ESR: erythrocyte sedimentation rate, IL-6: interleukin-6, NLR: neutrophil-to-lymphocyte ratio, CO-RADS: coronavirus disease 2019 Reporting and Data System, PCR: ploymerase chain reaction, CRP: C-reactive protein

Parameter	Percentage/ mean and standard deviation	Range
RT-PCR status		
Positive	1571 (67.05%)	
Suspect	772 (32.95%)	
CO-RADS		
CO-RADS 4 (high)	150 (6.40%)	
CO-RADS 5 (very high)	622 (26.55%)	
CO-RADS 6 (very high with PCR)	1571 (67.05%)	
CT severity score		
Normal	147 (6.27%	
Mild	724 (30.90%)	
Moderate	903 (38.54%)	
Severe	569 (24.29%)	
CRP positive (normal range, 0.00–0.50 mg/L)		
Normal	279 (11.91%)	
Mild	374 (15.96%)	
Moderate	1200 (51.22%)	
Severe	428 (18.27%)	
Not done	62 (2.65%)	
Lab parameters		
Ferritin (ng/ml)	321.83 ± 266.42	0 to 1650
LDL (U/L) (normal range, 125–220 U/L)	471.55 ±290.58	45 to 3875
ESR (mm/h)	53.6 ± 44.03	5 to 509
D-Dimer (mg/l or ng/ml)	1.51 ±0.85	0.1 to 3.9
IL-6 (pg/ml)	323.05 ±95.52	110 to 480
NLR (pg/ml)	8.4 ± 5.46	1 to 23.2
Hospital stay (in days)	10.25 ± 6.52	1 to 41
Disease outcome		
Deceased	417 (17.80%)	
Survived	1926 (82.20%)	

**Figure 1:**
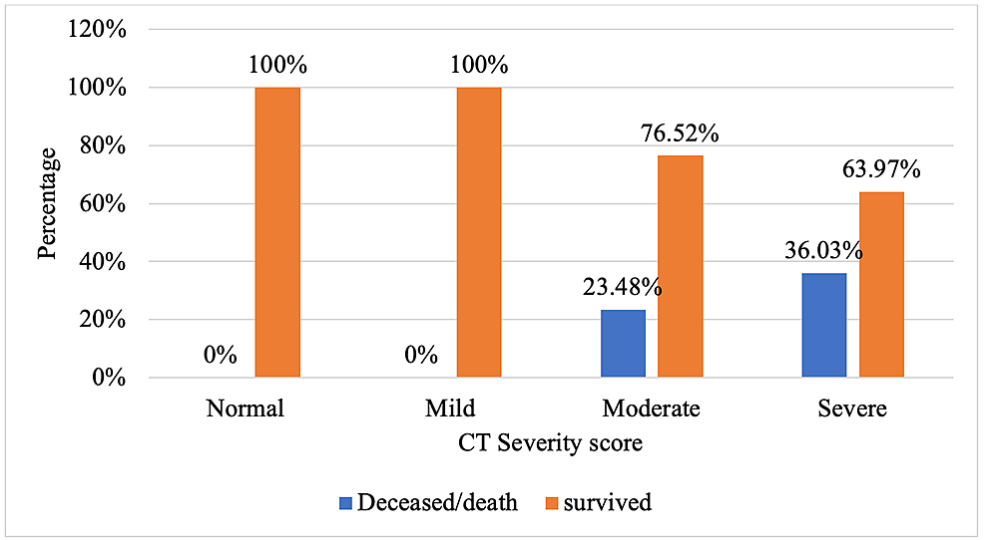
Clustered bar chart for comparison of CT severity score with disease outcome (N=2343) No statistical test was applied due to 0 subjects in the cells

All the participants with normal and mild CT severity scores survived. Out of 2343, 903 had moderate CT severity scores, 212 out of 903 (23.48%) participants died due to COVID complications, and 691 out of 903 (76.52%) survived. Out of 2343, 569 participants had severe scores, out of which 205 (36.03%) died, and 364 (63.97%) participants survived (Figure 1).

**Figure 2:**
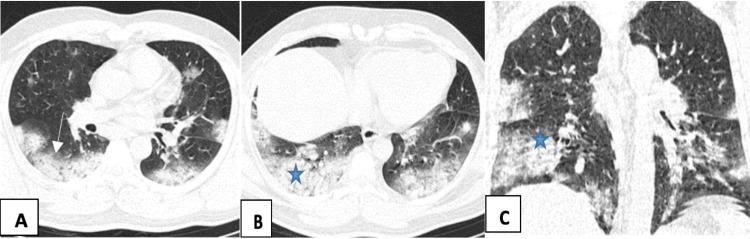
HRCT thorax axial (A&B) and coronal (C) reformatted images of a 70-year-old male patient demonstrates a few patchy areas of peripheral ground-glass opacities (long arrow) in both the lung fields with a CT severity score of 6/25 (mild disease)

Figure 2 shows HRCT thorax axial and coronal reformatted images of a 70-year-old male patient demonstrating a few patchy areas of peripheral ground-glass opacities in both the lung fields with a CT severity score of 6/25 (mild disease).

**Figure 3:**
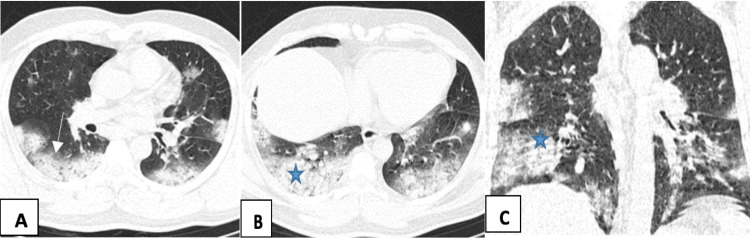
HRCT thorax axial and coronal reformatted images of 60-year-old male patient demonstrate a few patchy crazy pavement appearance (white arrow) and multiple peripheral areas of patchy consolidations (blue star), predominantly involving bilateral lower lobe with a CT severity score of 11/25 (moderate disease)

Figure 3 shows HRCT thorax axial and coronal reformatted images of a 60-year-old male patient demonstrating a few patchy crazy pavement appearances and multiple peripheral areas of patchy consolidations, predominantly involving bilateral lower lobe with a CT severity score of 11/25 (moderate disease).

## Discussion

This study from India is the first study that correlated CT severity scoring on HRCT thorax and inflammatory markers with COVID-19 related mortality in a designated COVID hospital. In the present study, more than half (1571, 67.05%) of the study population were symptomatic COVID-19 positive patients. We achieved high diagnostic performance results using CT scans with CO-RADS. The present study's authors followed the CT severity score part of CO-RADS. Most of them had a CO-RADS score between 4 and 6. The CT severity score was moderate in 903 (38.54%) and severe in 569 (24.29%) patients. Most (1926, 82.20%) of the participants survived, and 417 (17.80%) participants succumbed to COVID-related mortality.

The mean age in the current study was 51.69 ± 16.02 years, and most of the study participants were male. In comparison to the current study, Khan et al. [15] found the mean age of the patients as 54.0 ± 14.0 years (range: 24-83 years). All the laboratory parameters like mean ferritin level, LDH levels, erythrocyte sedimentation rate, D-dimer, interleukin-6, and neutrophil-to-lymphocyte ratio were highly altered in the present study. Kuzan et al. [16] found increased lactate dehydrogenase, C-reactive protein, and neutrophil-lymphocyte ratio in both laboratory-confirmed and clinically diagnosed COVID-19 groups. The finding was also compared to another study by Salvatore et al. [17], who found significantly higher CRP, leukocyte count, neutrophils, LDH, D-dimer, troponin, creatinine, azotemia, alanine aminotransferase (ALT), aspartate aminotransferase (AST), and bilirubin values.

In the present study, the majority (1571, 67.05%) had a CO-RADS score of 6, and only 150 (6.40%) had CO-RADS score of 4. Çomoğlu et al. [18] found 444 (33.1%) of the patients in the CO-RADS 1-2 and 894 (66.9%) in the CO-RADS 3-5 group out of 1338 patients similar to the present study. Our results indicate that a CO-RADS score ≥4 seems to ascertain the diagnosis of COVID-19. Therefore, a score of ≥4 can be used to put a patient in isolation or self-quarantine.

The CT severity score (CTSS) was average in only 147 (6.27%) participants and severe in 569 (24.29%) participants. The CTSS does not capture complications like pulmonary embolism, which increases the mortality rate of COVID-19. Similarly, Liefeld et al. [19] found that upper and lower CT score values help clinicians provide faster and timely management decisions in predicting patient disease course. A previous study by Li et al. [20] found that CTSS in asymptomatic patients (e.g., silent hypoxia) helps in monitoring and managing disease severity at admission. Lessmann et al. [21] found discriminated high performance between patients with COVID-19 and those without COVID-19 using CO-RADS and chest CT severity score assessed using artificial intelligence.

Using CO-RADS, an intuitive and reproducible standardized scheme for logging lung involvement related to SARS-CoV-2, radiologists have demonstrated their effectiveness and reliability in recognizing the classic HRCT appearances of COVID-19. According to the present study's findings, radiologists can use a definite reporting scheme in the early assessment of COVID-19 pneumonia on HRCT.

Indeed radiologists, are the first healthcare providers to recognition of HRCT thorax appearances consistent with COVID-19, even in asymptomatic subjects, is essential to limit the spread of the virus and prevent its transmission within hospitals, directing patients with high suspicion of COVID-19 pneumonia on HRCT thorax into the most appropriate care pathway. Prompt recognition of HRCT findings related to COVID-19 is fundamental in identifying patients who require comprehensive management in an Emergency Department setting, when RT-PCR test results are still pending, or in asymptomatic subjects. As the first healthcare personnel to evaluate chest HRCT images, Radiologists may have a pivotal role in this setting.

Strength: Both RT-PCR and CT scans for all the patients evaluated at our centre is the main strength of our work. The large sample size reflected the actual population for whom CT imaging was recommended.

Limitations: This study had several limitations. The present retrospective study was conducted at a single centre, so the findings cannot be generalized. The diagnostic criteria for COVID-19 were based on RT-PCR test which may have false negatives or false positive cases. The present study did not compare CT image findings and disease prognosis. Other upper and lower respiratory tract infections that show similar CT patterns similar to COVID-19 were not assessed.

Future perspectives: Further prospective multicentric studies covering a large geographical area are recommended to validate the findings of present study. is needed as to whether CO-RADS is useful for diagnosing COVID-19 in non-endemic areas.

## Conclusions

In conclusion, the present study results indicate a positive correlation of CT severity scoring on HRCT thorax and inflammatory markers with COVID-19 related mortality. The CT severity score, CO-RADS, levels of inflammatory markers increased with disease severity. Our findings support the use of CO-RADS and CTSS in early diagnosis and timely management of patients with COVID-19. Radiologists have a primary role in managing and caring for COVID-19 suspected patients. Applying this CO-RADS assessment system, typical HRCT imaging features involving lungs can be identified accurately and promptly.

## Appendices

The questionnaire consisted of the following questions:

a. What is your Age?

b. What is your gender?

c. Are you a health care worker?

d. Do you have any history of diabetes?

e. Do you have any history of hypertension?

f. Do you have any history of asthma/COPD?

g. Any known cardiac disease?

h. What is your vaccination status?

i. Any symptoms related to COVID-19 infection?

j. What was the RT PCR test status for COVID-19 infection?

k. What was the CT severity score?
